# ROSEBUD: A Deep Fluvial Segmentation Dataset for Monocular Vision-Based River Navigation and Obstacle Avoidance

**DOI:** 10.3390/s22134681

**Published:** 2022-06-21

**Authors:** Reeve Lambert, Jalil Chavez-Galaviz, Jianwen Li, Nina Mahmoudian

**Affiliations:** The School of Mechanical Engineering, Purdue University, West Lafayette, IN 47907, USA; lamber53@purdue.edu (R.L.); jchavezg@purdue.edu (J.C.-G.); li3602@purdue.edu (J.L.)

**Keywords:** semantic segmentation training dataset, unmanned surface vehicle, obstacle detection, deep learning, computer vision

## Abstract

Obstacle detection for autonomous navigation through semantic image segmentation using neural networks has grown in popularity for use in unmanned ground and surface vehicles because of its ability to rapidly create a highly accurate pixel-wise classification of complex scenes. Due to the lack of available training data, semantic networks are rarely applied to navigation in complex water scenes such as rivers, creeks, canals, and harbors. This work seeks to address the issue by making a one-of-its-kind River Obstacle Segmentation En-Route By USV Dataset (ROSEBUD) publicly available for use in robotic SLAM applications that map water and non-water entities in fluvial images from the water level. ROSEBUD provides a challenging baseline for surface navigation in complex environments using complex fluvial scenes. The dataset contains 549 images encompassing various water qualities, seasons, and obstacle types that were taken on narrow inland rivers and then hand annotated for use in semantic network training. The difference between the ROSEBUD dataset and existing marine datasets was verified. Two state-of-the-art networks were trained on existing water segmentation datasets and tested for generalization to the ROSEBUD dataset. Results from further training show that modern semantic networks custom made for water recognition, and trained on marine images, can properly segment large areas, but they struggle to properly segment small obstacles in fluvial scenes without further training on the ROSEBUD dataset.

## 1. Introduction

Unmanned Surface Vehicles (USVs) are a class of versatile mobile robots for autonomous navigation in water. USV applications range from blue water [1] and shallow waters [2] to slow moving inland waterways [3]. The use, design, and control of USVs in open environments is well studied and established in the literature. Simple path generation and following through basic control methodologies work well in such scenarios when an obstacle free a priori assumption is valid. However, for a USV to be practical for unsupervised use in dynamic water environments such as harbors, canals, and rivers, robust and near real-time environmental sensing and obstacle detection is required.

Non-navigable inland waterways are an example of a dynamic environment where most assumptions prior to operation will likely not hold, and a vehicle must perceive and understand its environment to update its path continuously to avoid obstacles and follow the river. River morphology [4] and storm debris can quickly make what limited maps exist of small river systems obsolete. Seasonal changes to water levels [5] also make navigation difficult, as the depth and course of the river may change daily, either exposing or submerging obstacles and barriers to travel. Both man-made structures and natural debris can also cause navigation problems and require detailed planning for avoidance. All of these barriers to autonomous operation create a fundamental issue facing autonomous navigation in inland waterways: with a lack of existing maps and a finite visual horizon, a robust perception method must exist to enable the Simultaneous Localization and Mapping (SLAM) of a river’s course and fluvial obstacles.

Many environmental sensing and obstacle detection methods from the ground domain have been implemented in the surface domain to provide capabilities of free space and obstacle detection. These methods typically involve ranging sensors such as LIDAR, RADAR, or visual sensors such as monocular or stereo cameras to gather high-dimensional data of the autonomous agents’ surrounding environment. However, due to the reflection and refraction of light at the water’s surface, and the cost of 3D LIDAR and RADAR modules, many recent works have focused on passive image-based approaches. This makes the obstacle and navigable space perception problem one of image processing and the recognition of water and objects in images.

Many image processing techniques are present in the robotics domain across all domains. Significant improvements have been made in the ground domain through the research in Unmanned Ground Vehicles (UGVs). Methods for image processing for robotic navigation typically fall into either traditional methods that use human selected features or convolutional network approaches that use trained encoders to extract features from images. Convolutional network approaches have shown excellent performance in the ground domain through deployment on UGVs by classifying items in environmental images. This classification can manifest itself through bounding boxes defining areas in images that contain objects of interest or through pixel-wise or semantic image segmentation whereby each pixel is given an associated class. The latter has shown an exceptional capability to not only recognize and classify objects present in images but also highly accurately locate objects, which is something that is desired for robotic applications.

Semantic classification enables a network to both classify and locate water within an image that contains both water and non-water regions. Just as in image classification, pixel-wise classification is performed by sequential convolution and pooling layers that summarize the contents of an image in a feature-rich latent space. However, unlike image classification, instead of flattening the latent space and utilizing fully connected layers and a soft-max function to create a label for the entire image, the latent space is either processed through super-pixel representations of the original image, a purpose-built decoder for the encoded latent space, or a fully convolutional neural network that performs inferences through subsequent kernels and deconvolutional steps.

Semantic segmentation networks have seen exceptional real-world use in autonomous ground vehicles such as autonomous cars but have seen very limited experimental validatation for use in marine environmental perception in harbors and littoral zones [6,7,8] and even less real world implementation [9] fo the navigation task. To the authors’ knowledge, semantic segmentation networks have not been explicitly applied to the river navigation and fluvial obstacle avoidance problem for USVs. This is in part because of the lack of publicly available annotated fluvial datasets that can be leveraged to train existing semantic networks. The River Obstacle Segmentation Enroute By USV Dataset (ROSEBUD) seeks to alleviate this data sparsity problem by providing the first of its kind publicly available dataset explicitly developed to train segmentation networks to recognize water in fluvial scenes.

The novelty of this work is thus in three parts: (1) The creation of the first of its kind fluvial segmentation dataset ROSEBUD with ground truth segmented images taken by the authors within fluvial scenes, (2) A study on the difficulty of the unique dataset by testing two state-of-the-art (SOA) water segmentation networks on ROSEBUD, and (3) A comparison of SOA methods and their ability to generalize to both fluvial and marine scenes.

In the remainder of this work, a review of methods for segmenting water and associated datasets is provided in Section 2. The ROSEBUD dataset is reviewed and detailed in Section 3. The experimental validation of the dataset on three semantic segmentation neural networks is detailed in Section 4. Finally, a conclusion and details on future work are provided in Section 5.

## 2. Related Work

Water recognition in images is an important aspect for both ground-based and unmanned surface vehicles. Ground-based vehicles must be able to recognize water for avoidance, while USVs must recognize water to determine navigable paths through mission areas. Throughout the literature, water recognition is performed using one of three methods: (1) algorithmic approaches that leverage hand selected image features, (2) traditional machine learning methods, and (3) Convolutional Neural Networks (CNNs).

Algorithmic approaches leverage assumptions about water and its surrounding environment to define rigid features such as image phase correlations [10], planes of reflection [11], and super-pixel reflectively and hue with linear binary patterns [12]. While these methods are exceptionally accurate for images that align with the a priori beliefs, they severely under perform when asked to perform in environments that exist outside of the a priori assumptions. Examples where certain assumptions may not hold include water on cloudy or dark days, water that is turbulent and thus does not have reflections, and rivers and lakes with poorly defined or shaded shorelines.

Traditional machine learning (ML) techniques used in water identification for USVs include decision forests [13] and Support Vector Machines (SVMs) [14]. While these methods generalize to varying conditions better than hand-crafted algorithms, they still struggle, as many of the features that machines are trained on are still hand selected, and forward inferences can be slow for high-dimensional feature spaces. Works such as [14] that leverage traditional machine learning techniques must use continuous unsupervised learning to achieve high accuracy (greater than 95%). This is both a time and energy-intensive task that is not well suited for rapid autonomous agent control.

Other methods such as active contouring modeling [15] have been used for image segmentation in addition to traditional ML-based approaches. These methods do not require significant a priori knowledge to find the boundary of water and have been used to segment lakes and other bodies of water in satellite imagery [16,17]. However, these methods struggle to rapidly reach solutions due to high computational loads and thus are not ideal for in situ ASV deployment. Recent works focus on improving the efficiency of such models through pre-fitting energy and adaptive functions [18] and by using additive bias methods [19]. However, even with these advances, the computational speed is generally still well below half a frame per second, proving dangerous to robotic operations using segmented outputs for navigational purposes. Therefore, active contouring modeling is not typically used for robotic applications, such as navigation, and it is not applied to the dataset presented in this work.

Image classification with CNNs can be further sub-categorized into work that leverages CNNs for either image classification, object localization, or semantic segmentation (pixel-wise classification) of water within images [20,21]. Advancements in semantic segmentation recently include Atrous convolution [22], Atrous Spatial Pyramid Pooling [22], Skip Layers that combine various resolutions of encoding and thus contain both dense features and location information [23], and residual layers [24].

Many recent advancements in semantic segmentation networks have made the networks easier to train, more generalizable, and better at resolving finer details for higher pixel-wise classification accuracy. This has made them the leading technique for visual perception for SLAM and robotic control. Significant work has been completed in the past decade to use semantic networks in the ground and air domain for self-driving cars and UAVs [25,26]. However, only recently have semantic networks been applied to water detection in robotic applications for the purposes of obstacle detection for USV avoidance [9], river level monitoring [20], and general USV SLAM/Control [6,7,8]. The lack of uptake in the use of semantic networks in marine domains is directly tied to the data-hungry nature of the networks themselves.

Most segmentation networks follow the supervised learning paradigm and thus require hand-annotated image masks that are used in loss function calculations. The masks are hand-annotated images that encode the ground truth information within an image. In general, the more data that are available for training and testing, the more generalizable a network can become. In the ground and air domains, a significant number of large publicly available and fully annotated datasets exist for the training of segmentation networks [27,28,29,30,31,32,33,34]. However, only a few small and publicly available annotated marine image segmentation datasets exist [6,9,35,36], and to the best of the authors’ knowledge, no publicly available semantically annotated datasets specifically designed for fluvial scenes as viewed on an USV on an inland river exist. Other works that have been completed with the specific intent of segmenting or identifying shorelines of fluvial scenes for USV navigation [10,11,12] do not use semantic networks and thus either have no associated datasets or did not publish their respective datasets. Other works for ground-level imaging of rivers for semantic segmentation such as [20] no longer have publicly available datasets, and works such as [9,37] either do not provide annotated data or have fluvial images that only contain the imagery of a single shoreline taken from the opposing shore, as would be present in large bodies of water.

## 3. ROSEBUD Dataset

To capture the realism and variance of images from a USV operating in fluvial environments, ROSEBUD [38] (see Data Availability Statement) consists of 549 images collected on two rivers in the US state of Indiana during the summer and fall of 2021 from a vehicle traversing the river. All video was recorded by the authors at 30 Frames Per Second (FPS) and with a resolution of 1920×1440 pixels from a GoPro Hero4 camera mounted approximately half a meter above the waterline.

A total of 249 unique image frames were extracted from over 2 h of video recorded on the Wabash River on 15 July 2021, while 300 images were extracted from over 3 h of video recorded on Sugar Creek on 30 September 2021. The Wabash River images were taken from a USV developed at Mahmoudian lab’s BREAM [39] that semi-autonomously traversed the Wabash River in Tippecanoe County. Sugar Creek images were taken from a Canoe that was piloted by a human operator over 2.5 h for 7.25 km between US Route 41 and B’Dale Road in Parke County. The two rivers were chosen because of the fluvial dichotomy between both river systems due to their differences in width, course, water clarity, obstacle types, sediment load, and environmental settings. While the dataset is gathered during the summer and fall months, augmentation can be performed on the dataset to present training data representative of winter and spring, as is discussed and performed on training data in Section 4.3.

**Figure 1:**
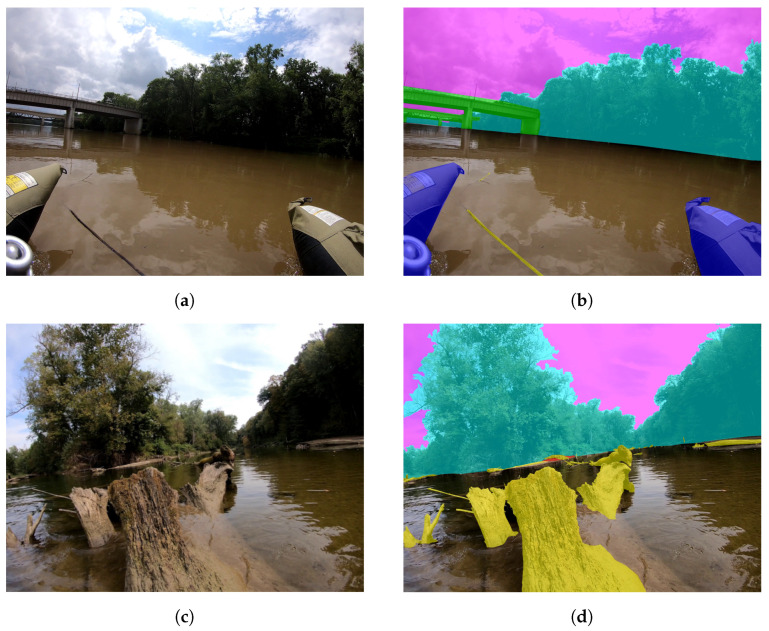
(**a**) Image captured on the Wabash River from a USV going through downtown Lafayette in Tippacanoe County, Indiana, (**b**) Annotation of (**a**) showing segmentation of the fluvial scene with annotations of bridge, sky, obstacles, and trees (river is disjoint of other classes), (**c**) Image captured from a canoe on Sugar Creek in Parke County, Indiana, (**d**) Annotation of (**c**) showing the annotation of the fluvial scene with annotations of the sky, trees, obstacles, and sandbar/riverbed [38].

The dataset contains three primary difficulties: (1) determining river shorelines at varying water levels and types, (2) identifying and segmenting navigation obstacles in the river, and (3) segmenting water when the river’s bottom and submerged obstacles can be seen through the water. The images from the Wabash River were taken during exceedingly high water, and they have no clear river shoreline evident due to sediment, rocks, or a change in flora. In contrast, images from Sugar Creek were taken during exceedingly low water levels, and they contain significant amounts of exposed river-bed. The latter problem is furthered by the water clarity, at times allowing visual imaging of the river bottom in the same image frame as the exposed river banks. The obstacles present on both rivers are also unique. The area of the Wabash River that was imaged does not have significant amounts of debris to avoid; the more urban nature of the river means that there are bridges and varying amounts of inorganic obstacles to avoid. As Sugar Creek runs through farmland, wooded areas, and alongside cliffs, it contains many boulders, rocks, downed trees, stumps, and exposed sand bars that must be segmented. An example from the Wabash River is shown in Figure 1a,b, while an example from Sugar Creek is shown in Figure 1c,d.

**Figure 2:**
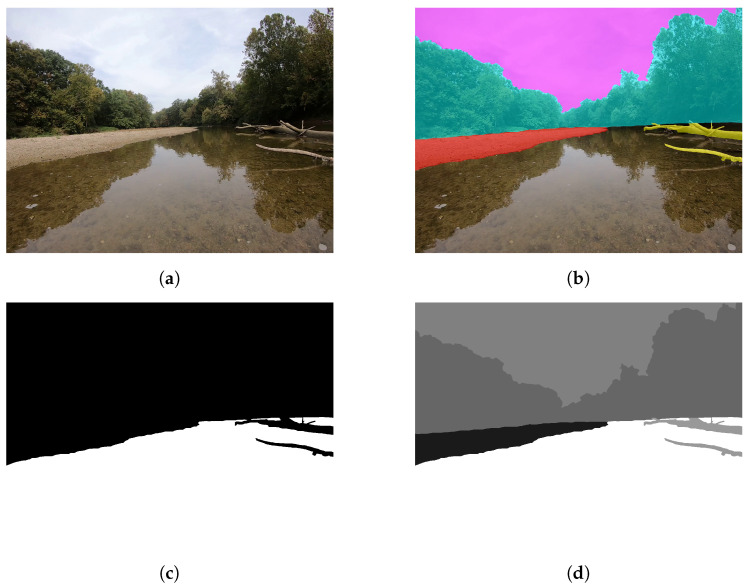
(**a**) An example of one of the 549 dataset images (**b**) Multi-Class Mask overlaid onto the image, (**c**) Binary classification mask for navigational purposes showing non-water and water segmented regions, (**d**) Multi-class segmentation mask [38].

All 549 images were manually annotated by human annotators utilizing a custom segmentation tool developed in Python that uses OpenCV to enable users to scroll through video frames, extract video frames, and augment the frames into trainable images. Annotation is carried out for seven fluvial classes: (1) water, (2) exposed river shore/bank, (3) bridge, (4) boat, (5) flora, (6) debris (logs, trash, rocks, debris), (7) sky, and then automatically saved as two masks and an over image as JPEG images. The first mask contains a label for all class regions as defined by the user as shown in Figure 2d; this is then overlaid onto the original image for user visualization and confirmation, as shown in Figure 2b. The second mask transforms the scene into a binary segmentation problem by concatenating all non-water classes together. Thus, the entire ROSEBUD set contains 4392 images with 549 original images, 549 binary classification masks, 549 seven-way segmentation masks, 549 ground-truth image labels, and all images/masks given at resolutions of both 1920×1440 and 512×384. In this way, the dataset can be utilized for training a network for two different tasks: identification of river paths for navigation and recognition of fluvial environment scenes for scientific surveys. While this work focuses on the binary classification problem for USV navigation, the fluvial segmented masks are provided in the public dataset for use by others.

**Figure 3:**
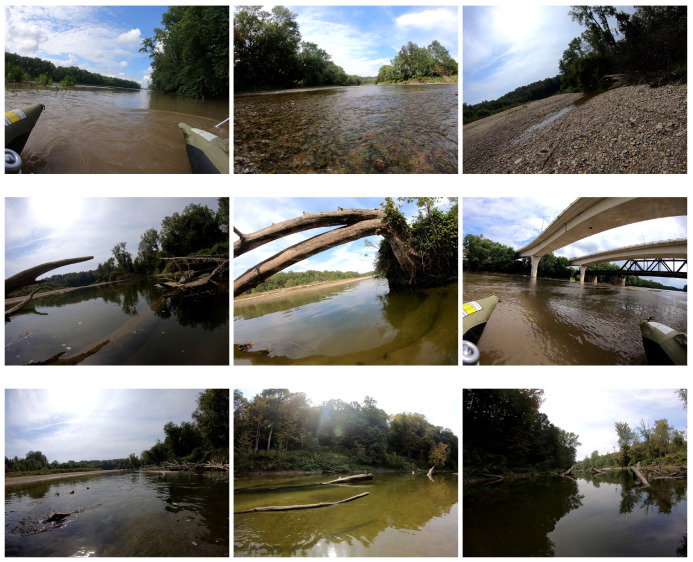
Examples from the ROSEBUD dataset [38] qualitatively showing variance in sizes of obstacles and scenery.

When studied from an obstacle identification perspective, the dataset stands in contrast to the current largest annotated segmentation marine dataset MaSTr1325 [6]. MaSTr1325 is dominated by obstacles in marine environments at large scales at an image resolution of 512×384. In comparison, ROSEBUD contains objects present in the rivers path on sizes of varying smaller scales at a resolution of 1920×1440 pixels. The obstacles present in the dataset also exist in many areas outside of the waterline, which poses a significantly more challenging localization problem for networks, as they must identify segmented class enclaves. Examples of obstacle and environment types are shown in Figure 3. Furthermore, unlike other publicly annotated water segmentation datasets that are dominated by the sea and sky, ROSEBUD also provides masks for seven unique fluvial classification providing a difficult classification task as well. In this regard, ROSEBUD provides data toward both river navigation and fluvial classification network development that the research community can leverage. A comparison between the ROSEBUD dataset and other current marine datasets is presented in Table 1.

To be as helpful to the AI community as possible, the ROSEBUD images and masks are given at a resolution of 512×384 in addition to the native 1920×1440 resolution. The masks for both image resolutions are also given in several different popular formats that can easily be utilized by segmentation network developers. The default binary masks are given as grayscale images with pixel representation of water (255) and non-water (0) given as 8-bit grayscale values. Binary masks are also given as 8-bit RGB images where the b-bit grayscale image is copied to each separate channel in the RGB image. Lastly, each binary mask is reported as a grayscale 8-bit image where the value of each pixel is tied to a class number of 1 or 0 for water and non-water, respectively. The fluvial masks are provided in a similar way as 8-bit RGB (3 channel) and grayscale (1 channel) masks.

## 4. Experimental Evaluation

To evaluate the novel need and difficulty of ROSEBUD, two current state-of-the-art segmentation architectures designed for water pixel classification in marine environments are used (Section 4.1). Each network is trained to classify water and non-water pixels on a combined dataset of the MaSTr1325 [6] and Tampere-WaterSeg [35,40] datasets, which together total 1925 images across several seasons and weather types. These images are then augmented to 26,950 images (Section 4.3). As both networks require different mask formats for training, the augmented image masks were turned into both three-channel 8-bit RGB masks (white and black for water and non-water) and 8-bit class identifier mask (pixel values of 1 or 0 for water and non-water pixels). After training each network for a number of epochs of the augmented dataset, the trained networks are tested on the ROSEBUD dataset binary classification masks (Section 4.4), and the performance is reported (Section 4.5) using the metrics defined in Section 4.2. Finally, the networks are trained for additional epochs on both ROSEBUD subsets (Wabash River and Sugar Creek) to identify if one type of fluvial environment would help the networks generalize to the other. All of the final results are detailed in Section 4.6.

### 4.1. Segmentation Architectures

Two state-of-the-art segmentation architectures are used to evaluate and baseline the ROSEBUD dataset. Both networks utilize a ResNet-101 backbone [41], with a fixed size CNN auto-encoder and a decoder back-end. Both of the selected networks have achieved high accuracy and F-1 scores on several existing marine datasets. While many other segmentation architectures exist including SEGnet [42], ESPNet [43], PSPNet [44], vanilla ResNet-101 [41], Fast FCN [45], and BiSeNet [46], only networks were chosen that were explicitly designed for, and evaluated on, the water recognition task. This way, the networks have the greatest chance of generalizing between marine datasets and the ROSEBUD dataset during testing.

#### 4.1.1. Water Segmentation and Refinement (WaSR)

WaSR [7,47] is a novel marine water semantic segmentation network that builds on the work of [6] and the analysis of different CNN architectures on the MaSTr1325 dataset. The WaSR encoder employs a segmentation backbone of ResNet-101 [41] in four residual blocks in combination with max-pooling layers and a hybrid atrous-convolution in the last two layers to increase the local spatial context. The decoder uses a combination of Attention Refinement Modules (ARFs) and Atrous Spatial Pyramid Pooling (ASPP) in parallel with a sequence of Feature Fusion Modules (FFMs) and ARMs that each take in residual spatial information from each residual block of the decoder. Training is performed with a novel Semantic Separation loss function that aides in the elimination of False Positives (FP) and False Negatives (FN). Finally, through an ASPP and softwmax layer, a color inference image is created. The network was trained on the MaSTr1325 dataset and tested on the MODD2 dataset. The network is present in a form that leverages in situ IMU information to infer the horizon and one that does not; this work utilizes the latter form.

#### 4.1.2. Water Obstacle Detection Network Based on Image Segmentation (WODIS)

WODIS [8] is a more traditional encoding and decoding framework that uses a U-Net inspired encoder–decoder structure to segment an input 512×384 image of marine scenes into water, sky, and obstacles. The encoder utilized is derived from the Xception network [48] that utilizes depth-wise separable convolutions in lieu of traditional convolution to extract deep features from the image. Three such convolutions are run in series and then run through an ARF; this is completed in two subsequent sub-stages that concatenate the dense information from the previous sub-stages at each separable convolutional layer, thus capturing the spatial data between each serialized convolution. The decoder works much the same way in reverse with FFMs taking the place of the convolutional block. The dataset was trained on the MaSTr1325 dataset and cross-referenced on the SMD, MID, and MODD2 datasets. The WODIS network can be trained with and without initializing the RESNET backbone with trained Xception model weights. Thus, to test the importance of pretrained weights on the fluvial water recognition learning task, WODIS was implemented both with and without the pretrained Xception weights provided by the authors.

### 4.2. Performance Evaluation Protocol

Performance of the networks in segmenting water in fluvial environments is completed through inference on ROSEBUD. The effectiveness and usefulness of the networks in USV fluvial navigation is reported with four scores relative to the binary segmentation task at hand:

- Specificity: $\frac{\mathrm{TN}}{\mathrm{TN}+\mathrm{FP}}$.
- F1 Score: $\frac{\mathrm{Pr}\times \mathrm{Se}}{\mathrm{Prc}+\mathrm{Se}}$ where Precision (Pr) and sensitivity (Se) are defined as: $\mathrm{Se}=\frac{\mathrm{TP}}{\mathrm{TP}+\mathrm{FN}}$ and $\mathrm{Pr}=\frac{\mathrm{TP}}{\mathrm{TP}+\mathrm{FP}}$, respectively.
- Mean Intersection Over Union: $\frac{\mathrm{TP}}{\mathrm{TP}+\mathrm{FP}+\mathrm{FN}}$.
- Mean Pixel Accuracy (Pa): $\frac{\mathrm{TP}+\mathrm{TN}}{\mathrm{TP}+\mathrm{TN}+\mathrm{FN}+\mathrm{FP}}$.

Here, *n* is the number of samples in ROSEBUD. TP, TN, FP, and FN are all the conventional terms for total dataset classification of True Positives (TP), True Negatives (TN), False Positives (FP), and False Negatives (FN). Other typical segmentation metrics such as a confusion matrix are not reported as this work, as it only deals with the binary semantic segmentation aspect of ROSEBUD.

Specificity was chosen to be explicitly reported for its importance to the navigation task of water segmentation. Having some water classified as non-water (FP) for navigation purposes is less detrimental to USV health than non-water classified as water (FN). Therefore, a tradeoff between specificity and accuracy is allowable for the networks. Pixel Accuracy (Pa) and F1-score are also given to provide insight into how well each network grasps river scenes in total. The Mean Intersection Over Union (MIOU) is reported as it gives insight into the overlap between binary classes at the water’s edge.

### 4.3. Data Augmentation

**Figure 4:**
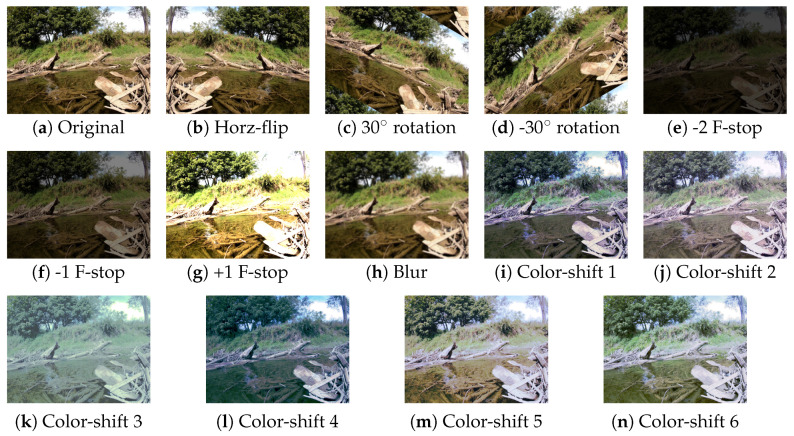
Each training image is augmented in 13 different ways as shown above. Example sequence uses images from Sugar Creek ROSEBUD Subset [38].

As water is exceptionally dynamic spatially and temporally in color, texture, and reflectively, care is taken to prevent overfitting to a specific time of day, season, or water state through augmentation. Augmentation is also completed to provide invariance to the USV position and thereby camera roll due to currents and waves. Dataset augmentation was carried out as shown in Figure 4, whereby each image in the original dataset is augmented 13 different ways from the original image, bringing the total training dataset size from 1925 to 26,950 images. Augmentation was completed independently of training with a developed augmentation tool that utilizes the skimage python library.

A horizontal flip and ±30 degree rotation was performed to provide orientation invariance. Three brightness augmented images are produced to provide incident light invariance by raising and lowering the F-stop of the image, where each ±n f stop is approximated by a channel pixel value change of $\pm 2^{n}$ capped at [0,255]. A Gaussian blur is added to the image to account for fast movements of water (including rain) and objects in static images. Finally, six color augmentations are completed to account for changes in how water hue can change with season, time of day, and sediment loading.

**Figure 5:**
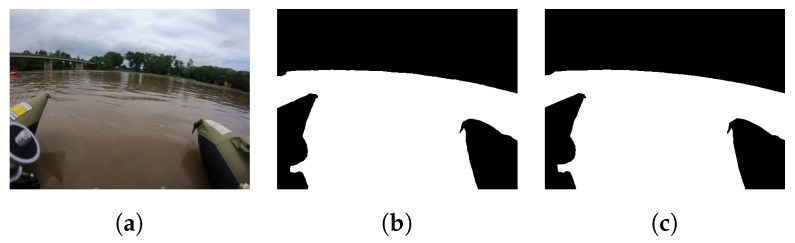

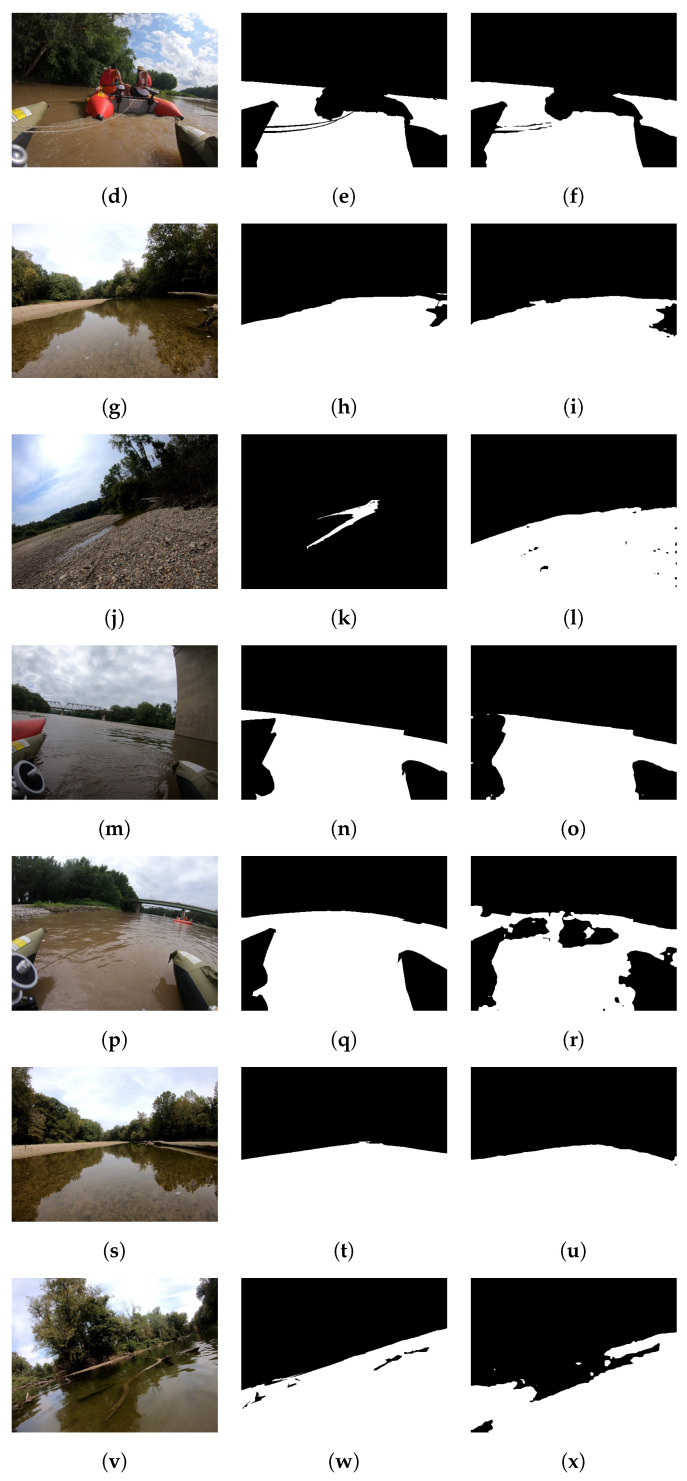
Qualitative outputs of Wasr and WODIS_Xcept networks on the ROSEBUD dataset [38] after completion of training as detailed in Section 4.4. From left to right, the columns show the test image, ground truth annotation, and the output mask by the network. Images (**a**–**l**) are WaSR Network Outputs, while (**m**–**x**) are WODIS_Xcept network outputs.

**Figure 6:**
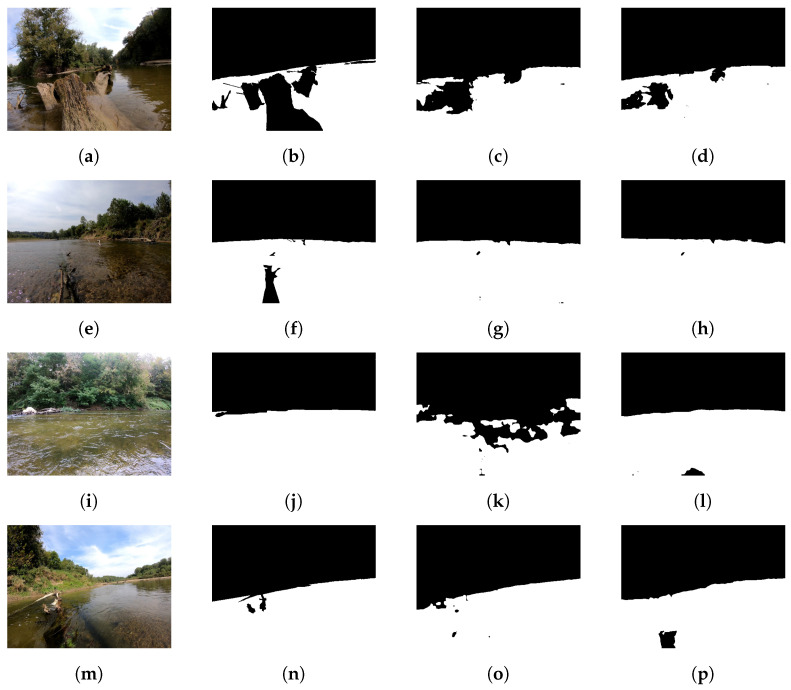
Illustration of network outputs on Sugar Creek scenes after further training on the ROSEBUD Wabash River image subset [38]. Images (**a**–**h**) are WaSR outputs after training 4 epochs on Wabash River images, and (**i**–**p**) are WODIS_Xcept network outputs after training an additional 20 epochs. Columns from left to right are the test image, the ground truth mask, the output of the network after initial training, and the output of the network after further training on the Wabash River dataset.

Image hue was changed by changing the original images color histogram to match that of what we call a “donor image”. The six donor images contain: (1) an image taken of a park in West Lafayette at sunset during “golden-hour” and thus containing redder light (Figure 4i), (2) an example image from the Tampere-WaterSeg open-water subset during overcast and light rain, resulting in diffused light (Figure 4j), (3) an over-saturated image taken from the MaSTr1325 dataset (Figure 4k), (4) an image taken on the water of Fairfield Lakes park in Tippecanoe County, Indiana, during winter (Figure 4l), (5) an image taken of the Wabash River at high noon in mid summer while the river is exceptionally sediment laden (Figure 4m), and (6) an image taken of Fairfield Lakes park in mid-summer in the afternoon (Figure 4n).

### 4.4. Network Implementation and Training

As the WaSR and WODIS networks were designed for inferences of the MaSTr1325 dataset (four classes), both networks had to be modified for use in a binary segmentation schema. The WaSR and WODIS network dataloaders were changed to accept the binary classification images made from the MaSTr1325 and Tampere-WaterSeg datasets. Outside of configurable parameters, no major modifications to either network beyond that which was published were made. All networks were trained for several epochs using an 80–20% training and validation split. Each network was trained on a different randomized subset of the augmented dataset (26,950 images) of size 21,560 images and validated on the remaining 5390 images. Both networks were trained with Pytorch API version 1.10 running on Python 3.8 using the best hyperparameters reported in each networks respective paper and code base. The batch sizes and epochs used throughout all training present in the work are summarized in Table 2.

Initial training of the WaSR network was completed over 20 epochs in total spread across a training session of 15 epochs and five epochs. Both training sessions had a batch size of 10 images. To resume the second training session, the checkpoint was loaded of the previous training run. Training took place on a NVIDIA RTX3090 with 24 GB of VRAM and clock speed of 1.4 GHz. The hyperparameters used for the optimizer were those reported in the WaSR publication and associated published code. The ResNet-101 backbone of WASR was initialized with pretrained weights available from the Pytorch API.

Initial training of the WODIS network was completed over 100 epochs with a batch size of 6. The network was trained on a NVIDIA RTX3080 with 8 GB of VRAM and a clock speed of 1.4 GHZ. The hyperparameters used for the training were as reported in the WODIS publication and associated code repository [8], except for the change in learning rate at 50 epochs, as performed in the author’s online repository linked to in [8]. Instead of jumping from 0.0001 to 0.003 as in the publication, the learning rate is halved after 50 epochs, as shown in Equation (1). This reflects the current state of the code in the WODIS repository as of April 2022. The WODIS training was repeated again in the same manner but with an initialized ResNet-101 backbone from the Xception weights provided in the WODIS GitHub Repository. This pretrained backbone network is referred to as WODIS_Xcept in subsequent sections of this work.

(1) $$lr_{old}=\left\{\begin{array}{c} \mathrm{if}\;\mathrm{epoch}\;<\;50:\;0.0001\\ \mathrm{else}:\;0.003 \end{array}\right.⟶lr_{new}=\left\{\begin{array}{c} \mathrm{if}\;\mathrm{epoch}\;<\;50:\;0.0001\\ \mathrm{else}:\;0.00005 \end{array}\right.$$

### 4.5. Network Architecture Benchmark of ROSEBUD

After training of the WaSR and WODIS networks on the augmented datasets, all three networks were baselined against the ROSEBUD dataset as a whole as well as the Sugar Creek and Wabash River subsets, all three of which are unseen by the network. The WaSR network outperformed WODIS in prediction accuracy on the ROSEBUD dataset and all three subsets. The WODIS network with pretrained weights (WODIS_Xcept) achieved better accuracy than the WODIS implementation initialized with random starting weights after being trained for the same amount of epochs. This is shown in Table 3.

The obstacle-ridden Sugar Creek data subset was harder for the networks to accurately process, with the Wabash data fairly closely approximating open water situations on USVs, as illustrated in Figure 5, and with all trained networks able to have an extremely high MIOU and MPA on the dastaset. Baselining on the entire ROSEBUD dataset worked well for both networks that were initialized with pretrained weights.

In general, it can be seen from Table 3 that each network, especially those with loaded pretrained weights, had quantitative results on the ROSEBUD dataset comparable to that reported in their respective papers where they were tested on the MaSTr1325 and/or SMD datasets. All networks have MPA and MIOUs in the mid 90s, with F1 scores in similar ranges. However, this differs from some of the qualitative results shown in Figure 5 and Figure 6 that show the network occasionally struggling with the more complex fluvial scenes that are ridden with small obstacles. We hypothesize this is due to three main factors: (1) while the small size of areas/obstacles to segment in the ROSEBUD dataset makes the dataset more challenging, the smaller obstacles contribute less to typical quantitative results, (2) while the dataset contains some images of complex shapes, other images lack such obstacles in ground truth masks and are exceptionally well segmented by the network increasing the network’s accuracy on the dataset as a whole, and (3) while aspects of the dataset are more challenging, the binary classification tasked used in this work is a simpler problem for the network architectures to learn over the multi-classification data they were built for.

The WODIS implementation that was initialized without pretrained weights significantly struggled on all datasets with MIOU that were always under 0.86. This reinforces the need for increased context recognition that is brought into the network by pretrained weights. Across all of the networks that were baselined, the MIOU was either marginally or significantly lower than the MPA. This in part due to the nature of the dataset and especially the Sugar Creek data subset, which contains many enclaves of segmented classes within areas of other classes (i.e., obstacles, rocks, logs, and branches).

The qualitative data for network accuracy shown in Figure 5 stand in contrast to the quantitative results of the baseline. With fluvial dynamic environments, the environment can change rapidly. In some instances, such as that shown in Figure 5j, the river can dry up or become extremely narrow, providing a scene that is unrecognizable to the network. In addition, while MPA can be high, the network may fail to recognize obstacles that suddenly enter the frame in both the foreground and the background at the same time, as shown in Figure 5v. While these and many smaller obstacles may not contribute highly to accuracy and other quantitative stats, they can prove detrimental to a USV navigating in such an environment. This is the fundamental challenge of the dataset. Images in open river environments such as Figure 5a,s,m are easily recognizable by all of the networks due to the similarities with the marine datasets the network was trained on. The largest issue for the networks in general is the reflections and accompanied drastic changes in the hue of the water in some images, as shown in Figure 5p.

### 4.6. Fluvial Training and Testing

After all three networks were trained and baselined against the subsets of the ROSEBUD datasets, they were further trained on both the Wabash River and Sugar Creek subsets of ROSEBUD and cross-validated against the other subset. For additional fluvial training, each network was initialized with the weights, as reported in Section 4.5 and trained for another four (WaSR) or 20 (WODIS) epochs using the same hyperparameters as detailed in Section 4.4. After the additional training was completed, the networks were baselined against the opposing subset of ROSEBUD. All 249 Wabash River and 300 Sugar Creek images were augmented as detailed in Section 4.3 into 3486 images and 4200 images, respectively, to be used for training.

Both versions of the WODIS network were trained for an additional 20 epochs with a batch side of 6 on the Sugar Creek and Wabash subsets of ROSEBUD separately. The WaSR implementation was also again trained in the same way but with a batch size of 10 and for four epochs. Quantitative results of all three networks after the additional training on the Wabash River subset and subsequent testing on the 300 Sugar Creek images are shown in Table 4. Results from Sugar Creek training and testing on the 249 Wabash images are shown in Table 5.

Table 4 shows that training on the Wabash subset aided in segmentation of the Sugar Creek data. All three network implementations increased the MPA of the Sugar Creek dataset between 3 and 1.8% and increases in the F1 scores on networks around 2%. The largest increase was in the MIOU with increases between 3.2 and 9%. This increase can be seen qualitatively in Figure 6a,i. While large-scale areas of water and non-water stayed largely the same, many artifacts and enclaves disappeared, and the waterline was cleaned up, producing cleaner masks. Furthermore, even for images that did not improve with further training such as Figure 6e,m the quality did not degrade at all. It is likely that this is due to the network being exposed to certain aspects of water recognition unique to fluvial environments such as reflections, shading of the river (non-uniform water brightness), and likely the curvature of the GoRro used for the ROSEBUD dataset. Of interest is the decrease in specificity across the board for both the WaSR and Wodis_Xcept networks.

**Figure 7:**
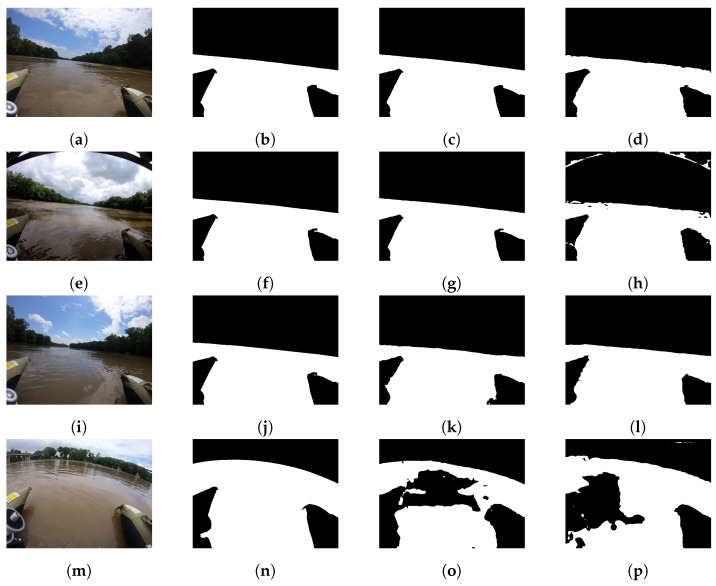
Illustration of network outputs on Wabash River scenes after further training on the ROSEBUD Sugar Creek image subset [38]. Images (**a**–**h**) are WaSR outputs after training 4 epochs on Sugar Creek images, and (**m**–**p**) are WODIS_Xcept network outputs after training an additional 20 epochs. Columns from left to right are the test image, the ground truth mask, the output of the network after initial training, and the output of the network after further training on the Sugar Creek dataset.

Qualitative results of the opposite training and testing procedure are shown in Table 5. Based upon the results, generalization appears to be only in a single direction for the ROSEBUD dataset. While large gains were made toward recognizing the Sugar Creek ROSEBUD subset through training on the Wabash River data subset, only minimal gains were made doing the opposite direction. For the most part, the F1 scores largely stayed the same or increased slightly. In the extreme example of the WODIS network, the F1 score actually decreased. The Mean Pixel Accuracy also remained largely unchanged. This is likely due to the difference in obstacle presence and camera position between the two datasets. As the Wabash images statically contain the pontoons of the USV used to collect that data, the network over-trained on the obstacle dense Sugar Creek subset and began to identify the reflections and hue variances as obstacles present in the image. This can be seen in Figure 7m.

This activity in cross-training and validation illustrates how well images from a navigable and obstacle-free river environment can aid network generalization to non-navigable and obstacle-filled fluvial scenes. The reverse is also true to identify if a network trained on obstacle dense scenes overfits and fails in more open areas. The approach to move from less to more densely cluttered scenes is completed in Table 4 and shown in Figure 4 works, as it follows a curriculum learning-based approach.

## 5. Conclusions and Future Work

In this paper, a first of its kind fluvial semantic segmentation dataset ROSEBUD was presented. The dataset aims to bridge the data sparsity problem that exists for marine systems and especially autonomous systems navigating in river environments and enable the use of supervised learning image segmentation techniques for navigation in fluvial applications. The dataset contains 549 hand-annotated binary class images that can be used to identify water in images as well as 549 images annotated with seven distinct fluvial classes.

To identify how different ROSEBUD is from existing marine segmentation datasets, two state-of-the-art semantic segmentation networks built for water recognition were trained on existing marine datasets. The trained networks were then tested to see how well they generalized to the ROSEBUD dataset and the Wabash River and Sugar Creek subsets. Both networks performed exceptionally well across the dataset as a whole, but they struggled with some images in the dataset that contain small amounts of water, dense obstacle scenes, or rapid changes in lighting from shadows. While such images do not detrimentally affect dataset performance, they can be detrimental for autonomous vehicles operating in dynamic environments. Further training networks on subsets of the ROSEBUD dataset helped both networks generalize to the fluvial environment, especially when pretrained weights were not incorporated into the network.

In the future, the ROSEBUD dataset will be used to fully train a lightweight segmentation network for implementation on a real USV platform for use in navigation river environments without any a priori understanding of its environment. In addition, the fluvial class masks can also be baselined and used for training with other network architectures to tackle more complex tasks. In the future, images can also be added to the dataset to provide data for fluvial scenes during winter months. The hope is that by making this dataset publicly available, it can add to the limited surface water annotated data and also allow others to train and develop networks for use in inland waterways.

## Figures and Tables

**Table 1:** Dataset comparison between MaSTr1325 [6], Waterline [9], Tampere-WaterSeg [35,40], and ROSEBUD.

Dataset	Resolution	Number	Classes	Environment	Types of Obstacle
MaSTr1325	512 × 384	1325	4	Coastal waters	Boats
Waterline	1920 × 1080	400	2	Lake and river	Buoys, sailboats, motorboats, and swans
Tampere- WaterSeg	1920 × 1080	600	2	Lake, canal, dock	Shoreline, docking areas, boats, rocks, maritime signs
ROSEBUD	1920 × 1440 512 × 384	549	2 and 7	River and creeks	Logs and branches, shore, fallen trees, rocks, sandbars, boats, canoes, and bridges

**Table 2:** Training parameters and weight initialization used for all the networks for each training session. Initial training is training the network on the augmented dataset as detailed in Section 4.3. The Wabash and Sugar Creek training each continue training for the epochs listed. During both instances, the networks are initialized with their respective weights from the initial training.

	Information			
	Model	Batch Size	Pretrained Weights	Epochs
Initial Training	WaSR	10	ResNet 101	20
	WODIS_Xcept	6	Xcept	100
	WODIS	6	Random	100
Wabash Training	WaSR	10	Base Training	4
	WODIS_Xcept	6	Base Training	20
	WODIS	6	Base Training	20
Sugar Creek Training	WaSR	10	Base Training	4
	WODIS_Xcept	6	Base Training	20
	WODIS	6	Base Training	20

**Table 3:** Quantitative comparison of networks after completing training on the augmented dataset as detailed in Section 4.3 and summarized in Table 2. Each network was baselined against the Wabash and Sugar Creek Subsets of the ROSEBUD dataset as well as the dataset as a whole.

**Network**	**MPA**	**MIOU**	**F1 Score**	**Spe**
Wabash River Stats after Initial Training				
WaSR	0.978	0.948	0.973	0.993
WODIS_Xcept	0.965	0.921	0.97	0.935
WODIS	0.929	0.842	0.94	0.872
Sugar Creek Stats after Initial Training				
WaSR	0.960	0.92	0.959	0.985
WODIS_Xcept	0.959	0.914	0.96	0.931
WODIS	0.929	0.855	0.936	0.871
ROSEBUD Stats after Initial Training				
WaSR	0.986	0.97	0.947	0.99
WODIS_Xcept	0.962	0.917	0.966	0.931
WODIS	0.9294	0.849	0.938	0.8714

**Table 4:** Quantitative capability of networks on segmenting the Wabash River subset of ROSEBUD after training on the Sugar Creek subset for the number of epochs as summarized in Table 2.

Wabash River Stats after Further Training				
Network	MPA	MIOU	F1 Score	Spe
WaSR	0.978	0.955	0.977	0.976
WODIS_Xcept	0.976	0.950	0.976	0.974
WODIS	0.959	0.916	0.960	0.942

**Table 5:** Quantitative capability of networks on segmenting the Sugar Creek subset of ROSEBUD after training on the Wabash River subset for the number of epochs as summarized in Table 2.

Sugar Creek Stats after Further Training				
Network	MPA	MIOU	F1 Score	Spe
WaSR	0.984	0.964	0.982	0.991
WODIS_Xcept	0.966	0.921	0.970	0.957
WODIS	0.925	0.844	0.934	0.954

## Data Availability

The dataset presented in this study is openly available in [River Obstacle Segmentation En-route By USV Dataset (ROSEBUD)] at https://doi.org/10.4231/MMJ2-NH88 (accessed on 29 April 2022).
